# Protein Engineering of Multi-Modular Transcription Factor Alcohol Dehydrogenase Repressor 1 (Adr1p), a Tool for Dissecting In Vitro Transcription Activation

**DOI:** 10.3390/biom9090497

**Published:** 2019-09-17

**Authors:** Memmo Buttinelli, Gianna Panetta, Ambra Bucci, Daniele Frascaria, Veronica Morea, Adriana Erica Miele

**Affiliations:** 1 Department of Biology and Biotechnology “Charles Darwin”, Sapienza University of Rome, P.le Aldo Moro 5, 00185 Rome, Italy; memmo.buttinelli@uniroma1.it (M.B.); ambra_bucci@libero.it (A.B.); daniele.frascaria@gmail.com (D.F.); 2 Department of Biochemical Sciences, Sapienza University of Rome, P.le Aldo Moro 5, 00185 Rome, Italy; giannapanetta24@gmail.com; 3 National Research Council of Italy (CNR), Institute of Molecular Biology and Pathology, P.le Aldo Moro 5, 00185 Rome, Italy; veronica.morea@cnr.it; 4 Institut de Chimie et Biochimie Moléculaires et Supramoléculaires (ICBMS), UMR 5246 CNRS–UCBL-Université de Lyon, 43 boulevard du 11 Novembre 1918, 69622 Villeurbanne, France

**Keywords:** Adr1p, transcription activation domains, multi-modular proteins, mini-gene design, structural bioinformatics, heterologous expression

## Abstract

Studying transcription machinery assembly in vitro is challenging because of long intrinsically disordered regions present within the multi-modular transcription factors. One example is alcohol dehydrogenase repressor 1 (Adr1p) from fermenting yeast, responsible for the metabolic switch from glucose to ethanol. The role of each individual transcription activation domain (TAD) has been previously studied, but their interplay and their roles in enhancing the stability of the protein is not known. In this work, we designed five unique miniAdr1 constructs containing either TADs I-II-III or TAD I and III, connected by linkers of different sizes and compositions. We demonstrated that miniAdr1-BL, containing only PAR-TAD I+III with a basic linker (BL), binds the cognate DNA sequence, located in the promoter of the *ADH2* (alcohol dehydrogenase 2) gene, and is necessary to stabilize the heterologous expression. In fact, we found that the sequence of the linker between TAD I and III affected the solubility of free miniAdr1 proteins, as well as the stability of their complexes with DNA. miniAdr1-BL is the stable unit able to recognize *ADH2* in vitro, and hence it is a promising tool for future studies on nucleosomal DNA binding and transcription machinery assembly in vitro.

## 1. Introduction

**Figure 1:**
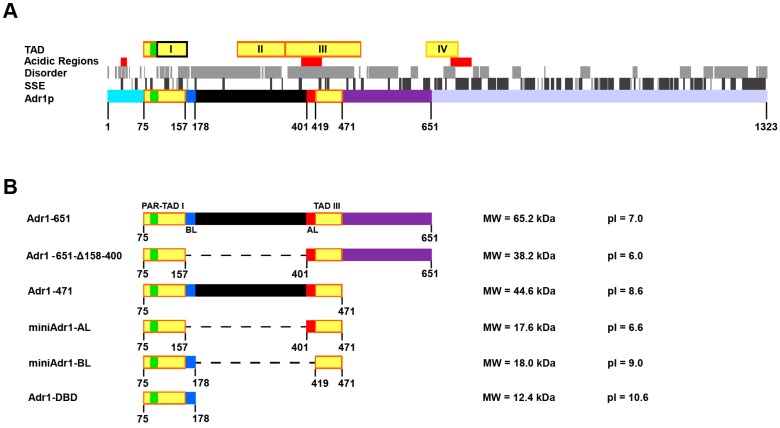
Adr1p and miniAdr1 constructs. (**A**) Schematic representation of full length Adr1p (UniProt ID: P07248, [27]). Acidic regions: residues belonging to the acidic regions, shown in red. Transcription Activation Domains (TAD) I, II, III, and IV are indicated by yellow boxes. The black square within TAD I indicates the largest Adr1p fragment, whose structure has been experimentally determined and is available from the PDB (PDB ID: 2ADR; [23]); the green box is the PAR region within TAD I. Segments of the protein between TAD I and TAD III are in black to highlight their deletion; residues of the basic linker and acid linker are in blue and red, respectively. Disorder: sequence regions predicted to be disordered by the PsiPred server [28], shown in light grey. SSE: Secondary structure elements, α-helices and β-strands, shown in dark grey and light grey, respectively. SSE in regions whose 3D structure has been experimentally determined were assigned by the PDBSum server [29], while in all other regions they were predicted by I-TASSER [30]. Adr1p: protein regions present in miniAdr1 constructs, indicated by different colors, with the number of starting and/or ending residues. (**B**) Schematic representation of miniAdr1 constructs following the color codes used previously (A). For each construct, the molecular weight (MW, kDa) and theoretical isoelectric points (pI), calculated by ProtParam [31], are indicated.

*Saccharomyces cerevisiae A*lcohol dehydrogenase repressor 1 (Adr1p, YDR216W) is the transcription activator of the *ADH2* gene (alcohol dehydrogenase 2) [1,2], which participates in the metabolic switch from glucose to ethanol or glycerol as food sources in yeast. Adr1p is involved in the activation of a number of genes of the respiratory metabolism, including those that regulate peroxisomes and phospholipid biosynthesis [3,4]. Depending on the type of fermentable or non-fermentable carbon sources present in the media, yeasts may switch metabolisms via Snf1p (the yeast homologue of AMP-activated protein kinase (AMPK) [5]) or by post-translational regulation of Adr1p activity [6,7]. When glucose levels drop, Adr1p is dephosphorylated and promotes the assembly of the transcription initiation complex by destabilizing two adjacent nucleosomes (−1 and +1), thus making the TATA box and the transcription starting site (TSS) accessible [8]. Therefore, Adr1p has been ascribed a dual role: (i) *ADH2* transcription activation, and (ii) repositioning of regulatory nucleosomes −1 and +1. Accessibility of the *ADH2* promoter to the micrococcal nuclease indicated that the *ADH2* gene is susceptible to chromatin remodeling in vivo [8,9]. This repositioning activity is not unusual, and it has also been demonstrated in vitro in TFIIIA, another zinc finger DNA-binding protein [10,11]. Adr1p recognizes and binds a 22-base-pair palindromic sequence of the gene *ADH2*, placed at −271 bases from the transcription start, called upstream activation sequence 1 (UAS1) [12]. UAS1 contains alternative A-T and G-C di- or tri-nucleotide repeats. Such repeats, which affect DNA bending and curvature, have been demonstrated to be important for nucleosome positioning and correct transcription activation [13,14,15,16,17,18]. At the primary structure level (Figure 1A), Adr1p is a complex protein made of 1323 residues, functionally arranged in four transcription activation domains (TADs). TAD I (residues 75–160) is the DNA binding domain (DBD) and contains two Cys2-His2 Zinc finger motifs (102–161), preceded by the so-called proximal accessory region (PAR) (residues 88–103). TAD II (263–357) and TAD III (359–509) are alternatively required for *ADH2* activation [12,19]. TAD I and TAD IV (642–704) directly contact TFIIB and TFIID [20,21], but not TBP. All TADs are reported to contact Ada2p and Gcn5p, both of which belong to the transcription activation complex [21]. The first 16 residues of Adr1p contain the nuclear localization signal (NLS). Three regions (29–40, 390–430, and 690–730) are rich in acidic residues, the second of which is at the N-terminus of TAD III [12,19,22] (Figure 1A).The aim of this work was to identify and produce in high yield a stable functional unit of Adr1p, able to bind cognate DNA, to be used in the future for in vitro biophysical studies of transcription activation and nucleosome repositioning. To this end, we designed, cloned, expressed, purified, and functionally characterized the miniAdr1 constructs listed in Figure 1B and Table 1.

All of the constructs contain TAD I and TAD III, but most of the regions between the two domains, as well as TAD II, were eliminated in some of them. As a control, we also cloned and expressed the DNA binding domain alone (Adr1-DBD, residues 75–178), a longer version of the solved structure of TAD I [23,24,25], which comprised only residues 102–161, 130–159, or 130–158. We believe that miniAdr1-BL will be a useful tool to be employed to better understand the assembly of the transcription initiation complex and nucleosome scanning in the presence of a specific transcription factor (TF), and not just of general TFs [26].

## 2. Materials and Methods

### 2.1. Design of the Minimal Regulatory Units

Design of miniAdr1 constructs was performed based on both data reported in the literature and results provided by programs to predict protein secondary and tertiary structures, as well as disordered or natively unfolded regions. The amino acid sequence of yeast Adr1p (UniProt ID: P07248, Adr1_YEAST, YDR216W) was downloaded from the UniProt database [27].

The Adr1p sequence was used to query freely available servers for the detection of homologous sequences or to predict 3D structures, protein domains, secondary structure elements, and disordered regions. In particular, we used: (i) the Blast and Psi-Blast programs [32] from the NCBI to detect proteins homologous to Adr1p in the non-redundant protein sequence database; (ii) I-TASSER [30] to perform predictions of secondary and tertiary structures and domain boundaries, since it consistently provided reliable protein structure predictions in community-wide Critical Assessment of Structure Prediction (CASP) experiments [33]; and (iii) PsiPred [28] Disopred3 program, one of the best at performing predictions of unstructured regions [34]. Assignment of secondary structure elements in the Adr1p region experimentally determined by NMR (2ADR, residues 102–160 [23]; 1PAA, residues 130–159 [24]) was derived from the PDBSum server [29].

### 2.2. Cloning and Expression

#### 2.2.1. Cloning

The designed miniAdr1 constructs were amplified by PCR from the full length gene coding for Adr1p inserted into pKD16 [35], using the primers listed in Supplementary Table S1. All the restriction enzymes and buffers were purchased by New England Biolabs (NEB, EuroClone, Pero, Italy). For prokaryotic expression, pET28b (Merck, Sial, Milan, Italy) was used. The constructs were inserted between *Nco*I and *Xho*I restriction sites, and a C-terminal His-tag was added. The *Pml*I and *Apa*I sites were used to express the designed constructs into the pPICZa vector (ThermoFisher Scientific, Rodano, Italy) of the fermenting yeast *Komagataella pastoris* (formerly known as *Pichia pastoris*) with a C-terminal His-Tag; the *Pml*I and *Xho*I sites were used to express the un-tagged proteins in the same expression host. The *BamH*I sites were inserted to ligate TAD I to TAD III. The starting DNA pieces were amplified by *Pfu* polymerase (Promega, Milan, Italy), following the manufacturer’s protocol. Each amplified product was conveniently digested with appropriate restriction enzymes and run on 2% agarose gel. Bands highlighted with ethidium bromide were excised under a UV trans-illuminator and purified with a QIAEX II gel extraction kit (Qiagen, Milan, Italy), following the manufacturer’s instructions. The pairs of fragments containing *BamH*I ends were ligated to obtain the designed miniAdr1 constructs before insertion into the expression vector. Ligation was carried out at 15 °C for 18 hours using T4-ligase (NEB, EuroClone, Pero, Italy). Linear DNA constructs were further purified on agarose gel and then inserted into the pPICZa vector, opportunely double-digested, and dephosphorylated with calf intestinal alkaline phosphatase (CIP; NEB, EuroClone, Pero, Italy). We obtained pPICZa-miniAdr1-BL and pPICZa-miniAdr1-AL with and without His-tag, pPICZa-Adr1-471 with His-tag, and pPICZa-Adr1-DBD without His-tag (Table 1, Figure 1B). All the constructs inserted into the plasmids were sequenced by Eurofins Genomics (Rome, Italy) and no variations were detected with the deposited sequence, aside from the deletions inserted on purpose.

#### 2.2.2. Expression in *Escherichia coli*

Heterologous expression in the prokaryotic host was performed by transforming BL21(DE3) competent cells (Agilent Technologies Italia, Cernusco sul Naviglio, Italy) with pET28b vectors containing the miniAdr1 sequences and His-Adr1-165. Expression was induced with 1 mM IPTG when liquid culture absorbance reached 0.5 at 600 nm, after being shaken at 180 rpm and 37 °C. Bacterial cells were then transferred to 18 °C for overnight expression of the proteins, having always been shaken at 180 rpm. The next day the cells were spun 10′ at 4000× *g* and the pellets were frozen at −20 °C until they were used for purification.

#### 2.2.3. Expression in *Komagataella pastoris*

The strategy used for *K. pastoris* expression was based on genomic incorporation of the desired gene sequences [36]. To this end, 5 μg of each plasmid produced for prokaryotic expression were linearized with *Xma*I before transforming the X-33 strain of *K. pastoris* (ThermoFisher Scientific, Rodano, Italy) by electroporation. Transformed cells were selected on YPD solid medium containing 100 μg/mL of Zeocin (ThermoFisher Scientific, Rodano, Italy) incubated at 30 °C for 48 h. The best expressing colony was chosen by transferring each colony in 5 mL Buffered Glycerol complex Medium for Yeast (BMGY) in 50 mL tubes. Colonies were allowed to grow for 24 h at 30 °C up to A_600_ = 5. Cells were then spun at 5000× *g* for 8 min, then the supernatant was discarded and substituted with Buffered Methanol complex Medium for Yeast (BMMY) to induce miniAdr1 construct expression. After 24, 48, and 72 h shaking at 200 rpm and 30 °C, aliquots were collected and centrifuged. Then, the pellets were checked for expression. The colonies showing the best expression were selected and stored in glycerol at −80 °C. For high yield production, 500 mL of transformed *K. pastoris* were grown in 2 L flasks at 28 °C, and shaken at 150 rpm in BMMY without methanol. After 24 h, 0.5% methanol was added to the medium and cells were harvested after a total of 48 h and stored at −20 °C for subsequent analysis.

### 2.3. Purification of miniAdr1 Constructs

#### 2.3.1. Purification of His-tagged miniAdr1 Constructs

All cell pellets were thawed in lysis buffer (20 mM Na/K phosphate buffer pH 6.9; 150 mM NaCl; 4 mM TCEP; 5 mM CHAPS; 5% glycerol; 1 mM benzamidine; 0.018 mM Zn Acetate; 1 mM PMSF) supplemented with one tablet of protease inhibitor (Roche, Milano, Italy). Cell lysis was mechanically performed on ice with glass beads (Merck-SIGMA Aldrich, Milano, Italy) in 1:1 volume (with respect to the lysis buffer). This disruption method was chosen because the amount of proteins was reduced by sonication (Supplementary Figure S1b, lanes 3–4). Beads were vortexed for 8 cycles of 30 seconds each. Debris were centrifuged for 5 min at 12,000× *g* and the pellets were discarded.

The supernatant was then filtered through a 0.45 μm syringe filter (Sartorius GmbH, Goettingen, Germany) and applied to a CM sepharose column equilibrated with lysis buffer containing 50 mM NaCl. After washing with 3 column volumes, a linear gradient was applied from 50 mM up to 1 M NaCl and fractions were collected and tested on SDS-PAGE and nucleoprotein polyacrylamide gel electrophoresis (N-PAGE) (also known as electrophoretic mobility shift assay (EMSA)) to check for the presence of pure and functional protein, respectively.

All His-tagged constructs expressed in *E. coli* or *K. pastoris* were also checked by Western blotting using anti-His-tag antibodies (Santa Cruz Biotechnology, Inc., Heidelberg, Germany).

In a first attempt to further purify His-miniAdr1-AL and His-miniAdr1-BL, fractions pulled from the CM or from Q sepharose were applied onto a HiTrap metal affinity column (GE Healthcare, Milano, Italia) loaded with NiSO_4_. Proteins were bound at 5 mM imidazole and purified with four steps at 60 mM, 100 mM, 300 mM, and 1 M imidazole. His-miniAdr1-AL and His-miniAdr1-BL were both eluted at 300 mM imidazole. However, the His-tagged proteins purified on Ni-NTA were not stable, and precipitation occurred when concentrated at more than 10 μM (Supplementary Figure S1e). This last point was the main reason for expressing the mini-genes without any tag.

#### 2.3.2. Purification of Untagged miniAdr1 Constructs

**Figure 2:**
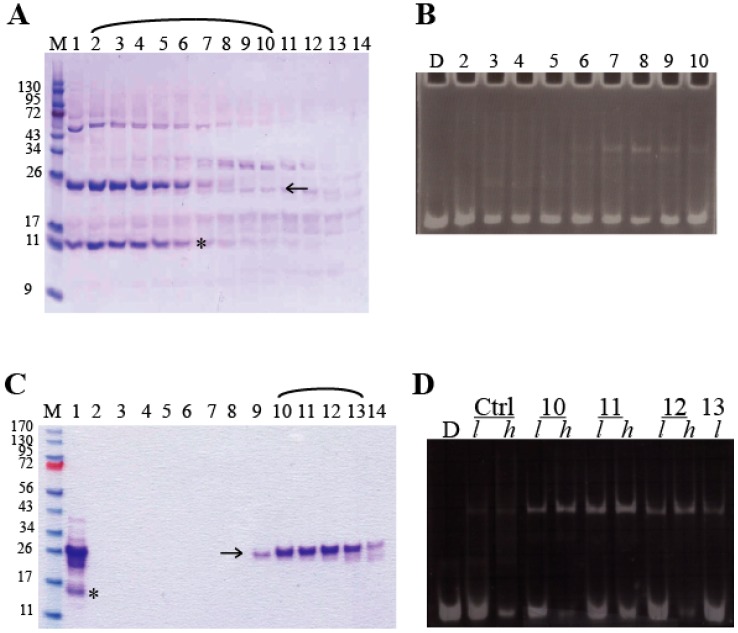
The best protocol of purification of untagged miniAdr1-BL. (**A**) The 4–12% gradient SDS-PAGE of the purification step on a CM sepharose column. Lane M: molecular weight markers (Fermentas, Thermo fisher Scientific, Milano, Italia). Lanes 1–14: fractions eluted at NaCl concentration between 0.5 and 1 M. Arrows and stars indicate miniAdr1-BL and KpCyt c, respectively. (**B**) Analytical DNA binding assays on fractions separated by CM sepharose (arch in Panel A). All fractions were reacted with half site dsDNA and stained with ethidium bromide. Lane D: free DNA. Lanes 2–10: CM column fractions corresponding to lanes 2–10 of Panel A. miniAdr1-BL is present in lanes 6 to 10. (**C**) The 12% SDS-PAGE after the second SEC-HPLC step. Fractions of CM column corresponding to lanes 6–10 of panel A were pulled, concentrated, and run twice on SEC-HPLC. Lane M: molecular weight markers (in kDa). Lane 1: input of the second HPLC step (coming from the peak of the first SEC). Lanes 2–14: fractions eluted from the second chromatographic step. (**D**) DNA-binding assay of the fractions separated by the second HPLC step (lanes 10–13 from panel C). Lowercase “l” and “h” refer to low (5 μL) and high (8 μL) volumes of protein solution in the binding assay. Lane D: half-site-free DNA probe. Lane Ctrl: previously purified miniAdr1-BL (positive control).

Untagged miniAdr1-AL, miniAdr1-BL, and Adr1-DBD were expressed, and the biomass was broken, as described above. The clear supernatant was applied to a CM–sepharose ion exchange column equilibrated with lysis buffer at pH 6.9, with 50 mM NaCl. A linear gradient from 50 mM to 1 M NaCl in lysis buffer was applied. This step yielded 70% pure proteins. The miniAdr1 constructs were co-purified with *K. pastoris* cytochrome c (KpCytc, Figure 2A) at about 500 mM NaCl. This event was particularly helpful, as KpCytc is red and miniAdr1-BL has only Phe and no Trp, which makes the chromatography detection at 280 nm harder. Fractions containing proteins were checked by SDS-PAGE for purity (Figure 2A) and N-PAGE (Figure 2B) for activity before they were pulled, concentrated, and further purified on two consecutive rounds of size exclusion chromatography (SEC Sephadex G-75, GE-Healthcare) on a HPLC apparatus (Lab Service Analytica, Anzola dell’Emilia, Italia). Since the molecular masses of miniAdr1 constructs and KpCytc (MW = 12 kDa; pI = 9.57) are similar, fractions enriched in miniAdr1 in a first round were concentrated again and re-applied to the same column to reach more than 90% purity (Figure 2C). The concentration of all the purified proteins was determined by UV-VIS spectrophotometry, using the sequence-based theoretical ε_280_ given by ProtParam [31].

### 2.4. Functional Characterization

#### 2.4.1. DNA-Binding Assays

The functionality of each construct during and at the end of the purification was assessed by N-PAGE-EMSA. Prior to protein binding, the oligonucleotides (Table 2) were allowed to anneal in order to produce a double stranded DNA. In fact, Adr1p binds only to dsDNA and not to ssDNA [12]. In the case of ethidium bromide detection, the protocol for oligo annealing was the following: either 800 pmol of palindromic ssDNA alone or 400 pmol of “half site a” plus 400 pmol of “half site b” (Table 2) were mixed in 20 mM Tris/HCl pH 8.2 in a final volume of 50 μL. In this way, 400 pmol of two sets of dsDNA were produced, the first one with 2 binding sites and the second one with only 1 binding site. The two sets were incubated at 80 °C for 5 min, then the entire block was left to cool down at room temperature.

The analytical binding reaction of the mini-constructs with DNA during purification was performed on the fractions coming from the chromatographic steps by using 6–8 μL of the eluate and 10^−8^ M cognate DNA, containing either the half site or the palindromic one. The reaction solution, totaling 20 μL, contained 10% glycerol, 18 μM Zn acetate, 5 mM DTT (or TCEP as reducing agent), 20 mM Na/K phosphate pH 7.5, 3.5 mM spermine, and 5 mM CHAPS (DNA-binding buffer). The use of such a mild zwitterionic detergent had the only purpose of keeping most of the protein in solution, thus rendering the recognition and binding more effective and concentration-dependent (Figure 2B,D). The proteins and the DNA were incubated for 30 min at 4 °C and then run on the gel.

In the case of radioactive detection, 200 pmol of the palindromic oligo to be labeled were mixed with 10 pmol/μL [γ^33^P]dATP at 3.3 pmol/μCi and 1 μL kinase (ThermoFisher Scientific) in 10 μL of kinase buffer. The reaction was incubated for 30 min at 37 °C, then transferred to ice, and 40 μL of ice cold 50 mM HEPES pH 7.5, 100 mM NaCl were added. The kinase was inactivated for 20 min at 65 °C and the mixture was loaded on a G50 spin column. At this point, the annealing step was started by adding 300 pmol of complementary oligo in 150 μL of 50 mM HEPES pH 7.5, 100 mM NaCl. As for the cold reaction, the DNAs were incubated at 80 °C for 5 min, then the entire block was allowed to cool down at room temperature. At the end, NaCl was added to the annealing mixture up to 300 mM, together with glycogen and ethanol; the DNA was precipitated at −70 °C and the salt was washed twice with 70% ethanol at 4 °C. The drained pellet was resuspended in 10 μL of 20 mM HEPES pH 7.5.

For the purified constructs, the DNA–protein binding was performed in 40 μL final volume. Then, 10 pmol of DNA were incubated with 40 pmol of each miniAdr1 construct in a binding buffer of 40 mM Na phosphate pH 7.5, 40 mM NaCl, 10% glycerol, and 18 μM Zn acetate. In some cases, reducing agents, mild neutral detergents, polyamines, and unspecific DNA were added to the mixture, as reported in the pertaining figure legends. Buffer and incubation times were the same as per the analytical binding.

The polyacrylamide gels for radioactive detection were cast at 4–6% (37.5:1 acrylamide/bis-acrylamide ratio) in the presence of 4–10% glycerol, with dimensions of 20 × 20 × 0.2 cm; 20–30 mM HEPES pH 7.5 was used as running buffer. The gels were run for 30 min at 4 °C and 5 mA during sample loading, and then at 10 mA and 120 V for 210 min. For the radioactive detection, the gels were dried prior to visualization by either autoradiography or with a phosphorimager. The gels for ethidium bromide detection of the DNA–protein complexes were cast at 7.5% acrylamide without glycerol; in this case, the running buffer was TB (45 mM Tris/boric acid) pH 8.3. The gels were run at room temperature at a fixed current of 7 mA and 90 V. For the ethidium bromide detection, the gels were incubated for 15 min in a solution of 0.5 μg/mL of ethidium bromide, then visualized under a UV trans-illuminator.

The DNA binding assays with constructs purified by HPLC were performed in 24 μL final volume. DNA and the proteins were mixed in 20 mM phosphate buffer pH 7.0, 40 mM NaCl, 10% glycerol, 35 μM Zn acetate, 5 mM DTT, 1 mM PMSF, 3.5 mM spermine, and 5 mM CHAPS.

#### 2.4.2. Quaternary Assembly by HPLC

Nucleoprotein complexes were pre-formed as described above and then run on a Superdex G75 prepacked column (void volume = 24 mL, GE-Healthcare) on a HPLC (LabAnalytical Service) equilibrated with 50 mM Na/K phosphate buffer pH 6.9, 150 mM NaCl, 4 mM TCEP, 5 mM CHAPS, 5% glycerol, 3.5 mM spermine, and 0.018 mM Zn Acetate. The flow rate was 0.4 mL/min and the complexes were injected in runs of 500 μL each. The column had been calibrated and the calibration curve used to calculate the corresponding molecular weights from the retention time was: log(MW) = −0.139·Vol_e_ + 6.16.

## 3. Results

### 3.1. Design of the Minimal Regulatory Units

To obtain functional protein fragments from multi-modular proteins, it is important to preserve the structural integrity of the domains of interest. Therefore, we searched the PDB [37] to obtain experimental information about Adr1p (UniProt: P07248) and proteins homologous to it. The 3D structures of the two Cys2-His2 Zn finger motifs located within TAD I of Adr1p were experimentally determined by NMR [23,24] and cryoEM [25]. Atomic coordinates are available from the PDB for residues 102–161 of Adr1p (PDB ID: 2ADR). Even though atomic coordinates for the N-terminal PAR region (residues 88–103) are not available, the Authors reported it to be unstructured in the absence of DNA, and to form a compact domain made by three antiparallel β-strands when bound to the UAS1 site [23,24,38]. Conversely, 3D structure information is not available for the remaining Adr1p regions (i.e., 1–101 and 162–1323), nor are present homologous proteins. Therefore, we queried bioinformatics servers able to provide reliable predictions of domains, secondary structure elements (SSE), and disordered regions. The results of the obtained predictions are reported in Figure 1A. Adr1p was predicted to contain large regions that do not assume a regular secondary structure (up to 72 consecutive amino acids), especially in the region between TAD I and TAD IV. This result is in agreement with the observation that intrinsically disordered regions are a common feature of proteins that need to assemble into large initiation complexes and contact many partners [39,40]. Conversely, most secondary structure elements (SSE) were predicted to occur after TAD IV in the C-terminal region of the protein.

The limited 3D structure information, low ratio between predicted SSE and disordered regions, and lack of domain definition contributed to making the design of miniAdr1 constructs endowed with DNA binding activity particularly challenging. For this reason, and to dissect the contribution of different Adr1p regions to protein stability and DNA binding activity, we designed five miniAdr1 constructs, shown in Figure 1B. All the miniAdr1 constructs include the PAR-TAD I regions responsible for UAS1 DNA binding (residues 75–157). These comprise both the two Zn finger motifs, which contact the DNA major grove of G-C rich regions, and the PAR, which also contacts the DNA major grove, but in correspondence with the A-T rich region, and which is required for high affinity DNA binding [23,41]. Additionally, they all contain a TAD III region (Figure 1B), which has been previously shown to be a strong activator of transcription in vivo [2,19,22], and contains a higher number of regions predicted to fold into SSE with respect to TAD II (Figure 1A). Indeed, previous studies have shown a functional redundancy of TAD II and TAD III [12,19]. However, we decided to shorten TAD III to include only the region with the highest density of residues predicted to assume a regular secondary structure.

Moreover, we chose as linkers either 20 residues at the C-terminus of TAD I (rich in basic amino acids, hence called basic linker (BL)) or 18 amino acids within the acid-rich region of TAD III (hence called acid linker (AL)), as reported in Table 3.

All other regions are different in the various constructs. In particular, Adr1-651 (comprising residues from 75 to 651) is a shorter variant of Adr1p containing the 3 TADs, and in which both N- and C-terminal regions, expected not to be involved in DNA binding, have been deleted. Two constructs derive from Adr1-651 by removal of further regions not expected to be essential in DNA binding: (1) Adr1-651_Δ158-400 (residues 75–157 are directly linked to residues 401–651), which lacks TAD II (predicted to be highly disordered); (2) Adr1-471 (residues 75–471), derived by removal of the C-terminal residues 472–651 after TAD III.

From this last construct the other three mini-genes are derived: (3) Adr1_Δ158-400 (residues 75–157 directly linked to residues 401–471), which carries both the deletion of TAD II and of the acid region linker; (4) Adr1_Δ179-418 (residues 75–178 directly linked to residues 419–471), which is a variant of (3) and links TAD I and TAD III via a basic rich linker, which is also predicted to have some secondary structure content (Figure 1A); and (5) Adr1-DBD (residues 75–178).

We shall refer now to construct (3) as miniAdr1-AL (acid linker) and to construct (4) as miniAdr1-BL (basic linker). The linker substitution from construct miniAdr1-AL to construct miniAdr1-BL has the effect of increasing the predicted isoelectric point from 6.6 to 9.0 (Figure 1B).

### 3.2. Cloning, Expression, and Purification of miniARD1 Constructs in Escherichia coli and Komagtaella pastoris

We first attempted to produce miniAdr1 constructs bearing a C-Terminal His-tag in *E. coli*, the most used bacterial system. Adr1-651, Adr1-471, and Adr1-651_Δ158-400 proved to be insoluble after expression (Table 1). Conversely, miniAdr1-AL and miniAdr1-BL presented slightly higher—but still limited—solubility, being only partially soluble when *E. coli* cells were grown at 18 °C.

Despite instability, aggregation, and purification problems, all proteins presenting a deletion of the internal TAD II region were functional (Supplementary Figure S1a). As a further control for DNA binding, we also expressed the His-tagged Adr1-165 [35], containing residues 1–165, cloned into pET28b, and comprising only TAD I, which did not purify to homogeneity (Supplementary Figure S1c,d).

To address the solubility problems, we expressed the miniAdr1 constructs in *K. pastoris*, a yeast cognate to *S. cerevisiae* (Adr1p source). *K. pastoris* is a methylotrophic organism able to express heterologous proteins in both the cytoplasm and extracellular environment. The constructs miniAdr1-AL and miniAdr1-BL were expressed both as C-terminal His-tagged and untagged proteins, while Adr1-471 was expressed with His-tag only, and Adr1-DBD was expressed without His-tag (see Section 2.2 and Table 1).

Concerning the yields, the best results were obtained by expressing the miniAdr1 constructs inside the cytoplasm, without His-tag, after 48 h induction (Table 1). The proteins purified by CM and Q sepharose columns and then applied onto an affinity chromatography were unstable and the yield was very poor (Supplementary Figure S1e and Table 1). This fact, together with the better solubility and stability of the untagged constructs, prompted us to continue the in vitro characterization only of the mini-constructs without any tags.

In order to purify the mini-constructs from *K. pastoris* cytoplasm, cells were lysed with glass beads, as sonication proved to be deleterious for the DNA binding activity (Supplementary Figure S1b). The purification was proceeded with cationic ion exchange chromatography (IEC), followed by different chromatographic steps for the different constructs (see Section 2.3). After each expression and purification step, the presence of functional protein was verified by DNA binding assays. An increase in terms of consistent quality and yield of purified miniAdr1-BL was achieved by using IEC followed by 2 rounds of size exclusion chromatography (SEC) on HPLC. In this way, the final yields allowed us to carry out a DNA binding assay with ethidium bromide staining (Figure 2B,D) instead of radioactive labeling.

In summary, the best overall results in terms of both heterologous expression levels, final purification yields, and purified protein stability were obtained with miniAdr1-BL without any tag (Table 1).

### 3.3. Functional Characterization of miniAdr1 Constructs

**Figure 3:**
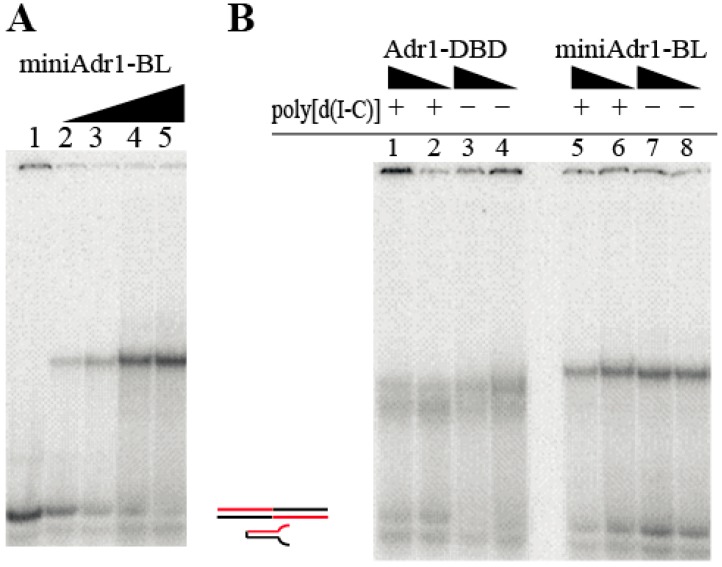
DNA binding assays of untagged miniAdr1-BL vs. Adr1-DBD assessed by N-PAGE/EMSA. Radioactive DNA probes were used. (**A**) DNA-binding properties of purified untagged miniAdr1-BL as a function of concentration in the presence of 5 mM CHAPS. Lane 1: free palindromic DNA probe (10^−8^ M). Lanes 2–5: palindromic DNA was incubated with increasing quantities of purified protein (3.16 × 10^−8^ M, 10^−7^ M, 3.16 × 10^−7^ M, and 10^−6^ M). The palindromic DNA runs as two bands: the upper is the complete dsDNA competent for binding; the lower is the hairpin DNA not competent for binding, where one strand was annealed on itself, because of the internal palindromic sequence (see cartoons on the right). (**B**) Effect of unspecific competitor [poly deoxy(I–C)] on palindromic DNA binding in the presence of 5 mM CHAPS. Plus (+) and minus (–) signs indicate the presence (40 pmol) or absence of the competitor. Two concentrations of protein were used: 40 and 20 pmol. Lanes 1–4: effect of unlabeled poly[d(I–C)] on Adr1-DBD binding to cognate palindromic DNA. Note that the protein–DNA population is not homogeneous (2 smeared bands) Lanes 5–8: effect of unlabeled poly[d(I–C)] on miniAdr1-BL binding to the palindromic DNA; no clear effect is visible.

**Figure 4:**
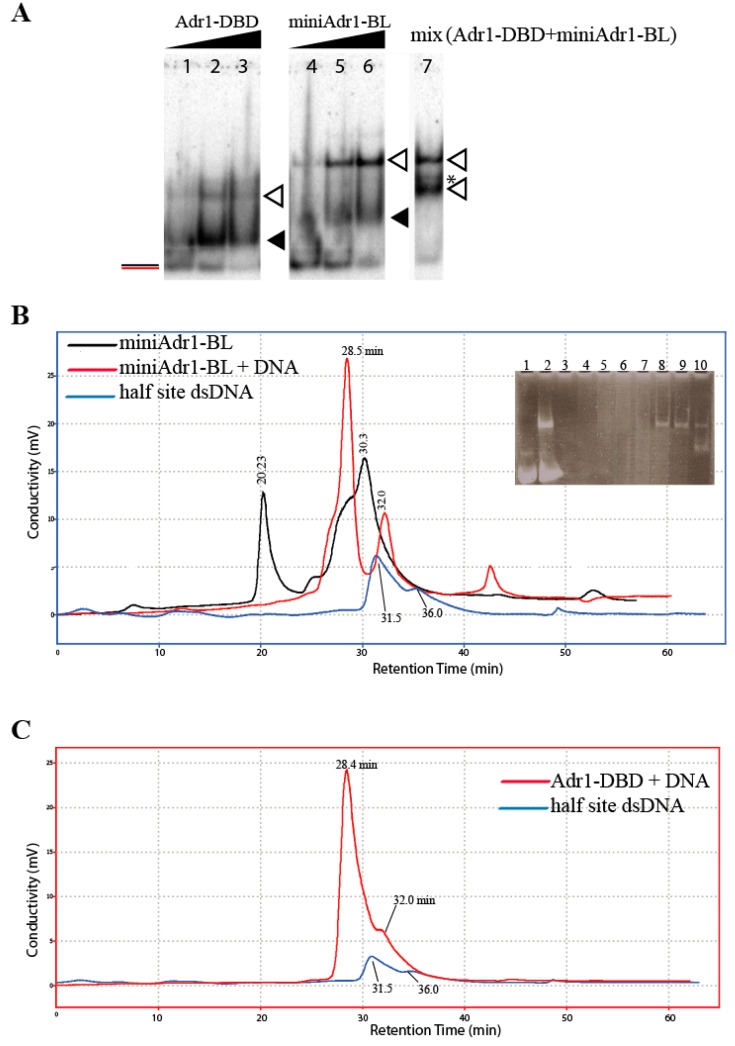
Quaternary assembly and stoichiometry of complex between miniAdr1 constructs and half site dsDNA, investigated with N-PAGE/EMSA (**A**) and SEC (**B**,**C**). (**A**) Effect of protein concentration on DNA binding in the absence of CHAPS and in the presence of half site dsDNA. Lanes 1–3: Adr1-DBD (20, 40, and 80 pmol). Lanes 4–6: miniAdr1-BL (20, 40, and 80 pmol). Lane 7: stoichiometric mixture of both proteins (40 pmol). The white arrowhead points to the homodimers, the black arrowhead to the monomers, and the asterisk to a putative heterodimer complex bound to the half site dsDNA. (**B**) Overlay of elution profiles of the half site dsDNA probe alone (blue chromatogram), miniAdr1-BL alone (black), and in complex with the half site dsDNA probe (red). Retention times (in minutes) corresponding to the peaks are marked. Insert: EMSA of SEC-collected fractions of the miniAdr1-BL+DNA complex (red chromatogram), revealed by ethidium bromide staining. Lane 1: fraction eluted at 32 min (free half site DNA probe). Lane 2: input to the gel filtration column for SEC experiment. Lane 3–7: fractions eluted between 20 and 27 min retention time. Lane 8: fraction eluted at 28 min. Lane 9: fraction eluted at 29 min. Lane 10: fraction eluted at 30 min. (**C**) Elution profiles of the half site DNA probe alone (blue chromatogram) in overlay with the profile of Adr1-DBD in complex with the half site DNA probe (red chromatogram). Retention times (in min) corresponding to the peaks are marked.

All of the purified miniAdr1 constructs produced in this study, either in prokaryotic or eukaryotic systems, are able to recognize and bind both the cognate palindromic sequence UAS1 of the *ADH2* gene, as reported for the full length wild type Adr1p [2,12], and the designed half site DNA at physiological pH (Table 2, Figure 2, Figure 3 and Figure 4, Supplementary Figure S1a–e). Untagged miniAdr1-BL presented a sharper band than untagged miniAdr1-AL (Supplementary Figure S1a,b).

miniAdr1-BL binds palindromic dsDNA in a concentration-dependent manner (Figure 3A), a feature clearly demonstrated by the increase of the signal of the shifted protein, and in parallel with the fainting of the upper band of free palindromic DNA. DNA binding is slightly affected by CHAPS (Figure 3 *versus* Figure 4A), while it is not affected by the presence of DTT as a reducing agent (Supplementary Figure S1b), indicating a correct folding of the Zn finger motif.

For comparison between our best construct (miniAdr1-BL) and the data in the literature, we also cloned Adr1-DBD and produced it in untagged form in *K. pastoris*, despite its intrinsic instability and non-homogeneous behavior in the DNA shift (Figure 3B). DNA binding of both constructs to palindromic DNA (which contains 2 binding sites) is not affected by the competition with unspecific poly[d(I–C)] (Figure 3B); however, miniAdr1-BL presents an overall sharper and neater band (Figure 3B lanes 5–8 *versus* 1–4). Indeed, this result suggested that miniAdr1-BL is the most stable minimal unit for binding of UAS1 DNA in vitro.

To analyze the behavior of miniAdr1-BL and Adr1-DBD in the presence of the half site cognate dsDNA, we used two independent techniques: band-shift assay on gel electrophoresis, and SEC on HPLC. The experiments depicted in Figure 4A show that the bands relative to DNA retardation are compatible with the presence of two populations of retarded molecules, which we attributed to monomers (black triangles) and dimers (white triangles). Adr1-DBD (Figure 4A, lanes 1–3) seems to prefer monomeric binding, while miniAdr1-BL binds mostly as a dimer (Figure 4A, lanes 4–6). We mixed together Adr1-DBD and miniAdr1-BL in stoichiometric concentrations (Figure 4A, lane 7). In the mixture, only the dimers were present (white triangles), suggesting that TAD III is a driver of oligomerization. Moreover, in lane 7, a third band is visible (marked with a star). We hypothesize that it might represent a heterodimer, given that the molecular weight difference between the two constructs is 6 kDa only (see Figure 1B). The presence of both monomers and dimers in a DNA binding assay with a half site dsDNA, which contains only one binding site, was also shown in partially purified His-tagged Adr1-165 expressed in bacteria (Supplementary Figure S1d lanes 12 to 14).

To gain further insight into dimer formation, we carried out experiments of SEC on HPLC on both miniAdr1-BL and Adr1-DBD (Figure 4B,C). When freshly purified and concentrated miniAdr1-BL (18 kDa) was applied to the column alone (Figure 4B, black chromatogram), the profile indicated an equilibrium of several forms. The sharp peak at 20.23 min is compatible with a pentamer or an aggregation of 5 monomers, whereas the broad peak at 30.3 min is compatible with a dimeric form (36 kDa). However, the latter peak contains a shoulder at 28 min, with retention times compatible with a trimeric form. One interesting feature arose when SEC was performed on the complex between miniAdr1-BL and half site dsDNA (12 kDa)—the peak corresponding to higher order aggregates completely disappeared (Figure 4B, red chromatogram). Moreover, the retention time of the first peak of the complex is compatible with a molecular weight of two proteins and one DNA molecule. The fractions eluted from the column between 20 min and 30 min were tested on acrylamide gel and stained with ethidium bromide (insert of Figure 4B). Indeed, the shoulders corresponding to retention times 20–27 min did not form a complex, while neat retardation was observed in fractions corresponding to a 2:1 protein/DNA ratio (lanes 8–9, retention times 28–29 min). The fraction at 30 min, represented by the valley of the main peak (see lane 10), contains a mixture of dimers and monomers. The final peak at 32 min contains free DNA only (lane 1, to be compared with the blue chromatogram in Figure 4B). When half site DNA is run alone (blue chromatogram in Figure 4B,C), it elutes in one peak at 31.5 min and a shoulder at 36 min. These retention times are compatible, respectively, with the formation of a concatenamer made by 2 dsDNA molecules and with one dsDNA alone. This is indirect proof that the designed overhang of the half site DNA might eventually be helpful in the crystallization of the complex.

Adr1-DBD (12.4 kDa), once bound to the same half site DNA, elutes as one very broad peak, with a summit at 28.4 min, broadening at 30 min, and displaying a shoulder at 32 min (Figure 4C, red chromatogram). The peak is compatible with 2 proteins and 1 DNA molecule (but also with 1 protein and 2 DNA molecules); the broader part is compatible with a 1:1 protein–DNA complex and the shoulder represents DNA alone (Figure 4C).

## 4. Discussion

The correct assembly of the transcription machinery at the right place and at the right moment is a crucial event in the life of every cell. This is even more evident in eukaryotic cells, where DNA is wrapped around nucleosomes, whose role goes beyond that of a simple scaffold. The entire transcription process is energy intensive, thus, it must be finely regulated to avoid unnecessary production of unwanted genes [26,42]. Over the years, it has been shown that many transcription factors are not just DNA binding proteins, but also effective multifunctional machines acting on DNA, nucleosomes (and hence on chromatin), and the transcription machinery by binding either co-activators or the polymerase itself. This functional plasticity is generally mirrored by a modular structure, whereby protein regions responsible for specific functions (either stably folded domains or intrinsically disordered domains able to fold upon binding to the right partner) are separated by intrinsically disordered regions. In most cases, the primary binding event is the recognition of cognate DNA sequences; once the first DNA-binding domain has “anchored” the DNA, intervening stretches can act as fishing lines, with one or more “baits” to recruit the rest of the machinery. In this way, transcription factors can act as sensors, regulators, and enhancers [11,39,42]. During the last thirty years, it has been shown that the 1323 residue transcription activator factor Adr1p also follows this mechanism [2,12,19,21,22,23,24,35]. In this work, our aim was to produce a stable, functional, minimal Adr1p region able to specifically bind DNA, and therefore, potentially capable of recruiting the transcription machinery and activating *ADH2* gene transcription. We demonstrated that miniAdr1-BL, comprising TAD I (PAR+DBD) and the core region of TAD III, linked by the basic stretch at the end of TAD I, is indeed able to specifically bind to the target DNA sequence, as predicted. Moreover, we have shown that the stoichiometry of the nucleoprotein complex is two proteins for each binding site on UAS1 of the *ADH2* gene, and that TAD III has an intrinsic capability to make protein–protein interactions with and without DNA (Figure 4B). This feature makes miniAdr1-BL able to form dimers both on palindromic and half site dsDNA (Figure 3 and Figure 4A), and also to form oligomers in the absence of DNA (Figure 4B). The fact that in the presence of the cognate DNA untagged miniAdr1-BL is mostly a dimer reinforces a previous hypothesis that TAD III is involved in the recruitment of transcription accessory proteins [22,35]. Furthermore, TAD I had been shown to bind both cognate dsDNA, TFIIB, and TFIID [20,21], thus, it contains a surface capable of protein–protein interactions, which we evidenced, unexpectedly, in the gel retardations as multiple bands, both in palindromic and half site DNA (Figure 3B and Figure 4A).

Interestingly, we found that the specific sequence connecting TAD I and TAD III (Table 3) has a significant effect on the stability of the purified constructs in the absence of DNA, since miniAdr1-AL, which contains a polar linker derived from the acid-rich region at the N-terminus of TAD III, is more prone to unspecific aggregation and precipitation than miniAdr1-BL, which contains a base-rich stretch present at the C-terminus of TAD I. Finally, we showed that the formation of a DNA–protein complex enhances the stability of all miniAdr1 purified constructs.

## 5. Conclusions

This is the first time (to the best of our knowledge) that a minimal unit of the transcription activator Adr1p containing just PAR-TAD I and TAD III, rather than only the Zn finger domains has been produced, purified to homogeneity, and used for the characterization of the stoichiometry of DNA binding in vitro. Despite a similar genetic construct, comprising residues 1–172 linked to 420–462 (i.e.,: NLS-PAR-TAD I and TAD III), was used in vivo to dissect the gene binding sequence and to map nucleosome shifting [12,35], neither of the two research groups proceeded further. We believe that the novel untagged miniAdr1-BL (comprising residues 75–178 linked to 419–471) implemented in this work will be an appropriate biotechnological tool to gain further insights about structure–function relationships in the assembly of the transcription machinery in vitro using single molecule biophysical techniques, and therefore, to study chromatin remodeling in vitro [10,26,43,44].

## Figures and Tables

**Table 1:** Final yields of miniAdr1 proteins produced in this study as functions of purified soluble proteins (see Figure 1B for their primary structure).

	*Escherichia coli* (His-tag)	*Komagataella pastoris* (His-tag)	*K. pastoris* (no tag)
Adr1-651	Insoluble (inclusion bodies, IB)	-	-
Adr1-651_Δ158–400	Insoluble (IB)	-	-
Adr1-471	Insoluble (IB)	insoluble	-
MiniAdr1-AL	<1 mg/L culture	<1 mg/L	1 mg/L unstable^1^
MiniAdr1-BL	<1 mg/L	<1 mg/L	3 mg/L
Adr1-DBD	-	-	1 mg/L unstable^1^
HisAdr1-165	~1 mg/L unstable ^1^	-	-

^1^ The notation “unstable” refers to the precipitation of the purified proteins at any of the following conditions: when concentrated >10 μM; after 1 week of storage at 4 °C; after 1 cycle of freeze-thawing at −20 °C.

**Table 2:** Sequences of oligonucleotides derived from UAS1 of the *ADH2* glucose-regulated gene and used in DNA binding assays. The sequence recognized by Adr1p wild type is in italics, while its palindrome is in bold. Once in double helix conformation, the first oligo has two binding sites, whereas the second has only one binding site, therefore, it was named the half site.

Oligonucleotide Name	Sequence
Palindromic	5′–CCGGCCTC*TCCAACTTA*·**TAAGTTGGA**GAGG–3′
Half site “a”	5′–TCCGGGCATC*TCCAACTTA*–3′
Half site “b”	5′–C**TAAGTTGGA**GATGCCCGG–3′

**Table 3:** Primary structure of the linkers between TAD I and TAD III in the miniAdr1 constructs. The numbers refer to the amino acid sequences in the full length protein (UNIPROT: P07248).

	Composition of the Translated Proteins with Respect to the Full Length Primary Structure	Linker Sequence
miniAdr1-AL	(75–157)–(401–471)	401–SWTVAIDNNSNNNKVSDN–418
miniAdr1-BL	(75–178)–(419–471)	158–NLGETISHTKKVSRTITKAR–178

## Supplementary Materials

The following are available online at https://www.mdpi.com/2218-273X/9/9/497/s1: Figure S1: Expression, purification, function, and stability of miniAdr1 constructs. Table S1. Sequences of oligonucleotides used as primers for PCR amplification of the mini-Adr1 constructs.

## Abbreviations

Ada2p, adenosine deaminase 2; *ADH2*, alcohol dehydrogenase 2 gene; Adr1p, alcohol dehydrogenase repressor 1 protein; BMGY, buffered medium glycerol-complex for yeast; BMMY, buffered medium methanol-complex for yeast; CASP, Critical Assessment of Structure Prediction; CHAPS, 3-[(3-Cholamidopropyl)dimethyl ammonium]-1-propanesulfonate hydrate; CM sepharose, carboxy-methyl sepharose; DBD, DNA binding domain; dsDNA, double stranded DNA; DTT, dithiothreitol; EMSA, electrophoretic mobility shift assay; GCN5p, general control of aminoacyl synthesis protein 5; HPLC, high performance/pressure liquid chromatography; IEC, ion exchange chromatography; KpCytc, *Komagataella pastoris* Cytochrome c; NLS, Nuclear Localization Sequence; N-PAGE, nucleoprotein polyacrylamide gel electrophoresis; PAR, proximal accessory region; PDB, protein data bank; PMSF, phenylmethylsulfonyl fluoride; SEC, size exclusion chromatography; ssDNA, single stranded DNA; SSE, secondary structure elements; TAD, transcription activation domain; TBP, TATA box binding protein; TCEP, Tris(2-carboxyethyl)phosphine hydrochloride; TF, transcription factor; TFIIB, transcription factor B of polymerase II; TFIIIA, transcription factor A of polymerase III; TSS, transcription starting site; UAS1, upstream activation sequence 1; YPD, yeast extract peptone dextrose medium.

## References

[1] Denis C.L., Ciriacy M., Young E.T. (1981). A positive regulatory gene is required for accumulation of the functional messenger RNA for the glucose-repressible alcohol dehydrogenase from *Saccharomyces cerevisiae*. J. Mol. Biol..

[2] Bemis L.T., Denis C.L. (1988). Identification of functional regions in the yeast transcriptional activator Adr1. Mol. Cell. Biol..

[3] Simon M.A., Rapatz W.G., Spevak W., Ruis H. (1991). The *Saccharomyces cerevisiae* Adr1 gene is a positive regulator of transcription of genes encoding peroxisomal proteins. Mol. Cell. Biol..

[4] Hahn S., Young E.T. (2011). Transcriptional regulation in *Saccharomyces cerevisiae*: transcription factor regulation and function, mechanisms of initiation, and roles of activators and coactivators. Genetics.

[5] Wierman M.B., Maqani N., Strickler E., Li M., Smith J.S. (2017). Caloric Restriction Extends Yeast Chronological Life Span by Optimizing the Snf1 (AMPK) Signaling Pathway. Mol Cell Biol..

[6] Blumberg H., Hartshorne T.A., Young E.T. (1988). Regulation of expression and activity of the yeast transcription factor Adr1. Mol. Cell. Biol..

[7] Taylor W.E., Young E.T. (1990). cAMP-dependent phosphorylation and inactivation of yeast transcription factor Adr1 does not affect DNA binding. Proc. Natl. Acad. Sci. USA.

[8] Verdone L., Cesari F., Denis C.L., Di Mauro E., Caserta M. (1997). Factors affecting *Saccharomyces cerevisiae* ADH2 chromatin remodelling and transcription. J. Biol. Chem..

[9] Abate G., Bastonini E., Braun K.A., Verdone L., Young E.T., Caserta M. (2012). Snf1/AMPK regulates Gcn5 occupancy, H3 acetylation and chromatin remodelling at *S. cerevisiae* ADY2 promoter. Biochim. Biophys. Acta.

[10] Panetta G., Buttinelli M., Flaus A., Richmond T.J., Rhodes D. (1998). Differential nucleosome positioning on *Xenopus* oocyte and somatic 5 S RNA genes determines both TFIIIA and H1 binding: A mechanism for selective H1 repression. J. Mol. Biol..

[11] Klug A. (2010). The discovery of zinc fingers and their development for practical applications in gene regulation and genome manipulation. Rev. Biophys..

[12] Thukral S.K., Eisen A., Young E.T. (1991). Two monomers of yeast transcription factor Adr1 bind a palindromic sequence symmetrically to activate ADH2 expression. Mol. Cell. Biol..

[13] Drew H.R., Travers A. (1985). DNA bending and its relation to nucleosome positioning. J. Mol. Biol..

[14] Battistini F., Hunter C.A., Moore I.K., Widom J. (2012). Structure-based identification of new high-affinity nucleosome binding sequences. J. Mol. Biol..

[15] Struhl K., Segal E. (2013). Determinants of nucleosome positioning. Nat. Struct. Mol. Biol..

[16] Di Marcotullio L., Buttinelli M., Costanzo G., Di Mauro E., Negri R. (1998). Changing nucleosome positions in vivo through modification of the DNA rotational information. Biochem. J..

[17] Buttinelli M., Minnock A., Panetta G., Waring M., Travers A. (1998). The exocyclic groups of DNA modulate the affinity and positioning of the histone octamer. Proc. Natl. Acad. Sci. USA.

[18] Negri R., Buttinelli M., Panetta G., De Arcangelis V., Di Mauro E., Travers A. (2001). Sequence dependence of translational positioning of core nucleosomes. J. Mol. Biol..

[19] Cook W.J., Chase D., Audino D.C., Denis C.L. (1994). Dissection of the Adr1 protein reveals multiple, functionally redundant activation domains interspersed with inhibitory regions: Evidence for a repressor binding to the Adr1c region. Mol. Cell. Biol..

[20] Hintze S., Engelhardt M., van Diepen L., Witt E., Schüller H.J. (2017). Multiple Taf subunits of TFIID interact with Ino2 activation domains and contribute to expression of genes required for yeast phospholipid biosynthesis. Mol. Microbiol..

[21] Chiang Y.C., Komarnitsky F., Chase D., Denis C.L. (1996). Adr1 activation domains contact the histone acetyl transferase GCN5 and the core transcriptional factor TFIIB. J. Biol. Chem..

[22] Thukral S.K., Tavianini M.A., Blumberg H., Young E.T. (1989). Localization of a minimal binding domain and activation regions in the yeast regulatory protein Adr1. Mol. Cell. Biol..

[23] Bowers P.M., Schaufler L.E., Klevit R.E. (1999). A folding transition and novel zinc finger accessory domain in the transcription factor Adr1. Nat. Struct. Biol..

[24] Bernstein B.E., Hoffman R.C., Horvath S., Herriott J.R., Klevit R.E. (1994). Structure of a histidine-X4-histidine zinc finger domain: Insights into Adr1-UAS1 protein-DNA recognition. Biochemistry.

[25] Nilsson O.B., Hedman R., Marino J., Wickles S., Bischoff L., Johansson M., Müller-Lucks A., Trovato F., Puglisi J.D., O’Brien E.P. (2015). Cotranslational Protein Folding inside the Ribosome Exit Tunnel. Cell Rep..

[26] Nagai S., Davis R.E., Mattei P.J., Eagen K.P., Kornberg R.D. (2017). Chromatin potentiates transcription. Proc. Natl. Acad. Sci. USA.

[27] The UniProt Consortium (2017). UniProt: the universal protein knowledgebase. Nucleic Acids Res..

[28] Buchan D.W., Minneci F., Nugent T.C., Bryson K., Jones D.T. (2013). Scalable web services for the PSIPRED Protein Analysis Workbench. Nucleic Acids Res..

[29] De Beer T.A., Berka K., Thornton J.M., Laskowski R.A. (2014). PDBsum additions. Nucleic Acids Res..

[30] Roy A., Kucukural A., Zhang Y. (2010). I-TASSER: A unified platform for automated protein structure and function prediction. Nat. Protocols..

[31] Gasteiger E., Hoogland C., Gattiker A., Duvaud D., Wilkins M.R., Appel R.D., Bairoch A., Walker J.M. (2005). Protein Identification and Analysis Tools on the ExPASy Server. The Proteomics Protocols Handbook.

[32] Altschul S.F., Madden T.L., Schäffer A.A., Zhang J., Zhang Z., Miller W., Lipman D.J. (1997). Gapped BLAST and PSI-BLAST: A new generation of protein database search programs. Nucleic Acids Res..

[33] Huang Y.J., Mao B., Aramini J.M., Montelione G.T. (2014). Assessment of template-based protein structure predictions in CASP10. Proteins.

[34] Monastyrskyy B., Kryshtafovych A., Moult J., Tramontano A., Fidelis K. (2014). Assessment of protein disorder region predictions in CASP10. Proteins.

[35] Di Mauro E., Kendrew S.G., Caserta M. (2000). Two distinct nucleosome alterations characterize chromatin remodelling at the *Saccharomyces cerevisiae* ADH2 promoter. J. Biol. Chem..

[36] Weidner M., Taupp M., Hallam S.J. (2010). Expression of recombinant proteins in the methylotrophic yeast Pichia pastoris. J. Vis. Exp..

[37] Burley S.K., Berman H.M., Kleywegt G.J., Markley J.L., Nakamura H., Velankar S. (2017). Protein Data Bank (PDB): The Single Global Macromolecular Structure Archive. Methods Mol. Biol..

[38] Hyre D.E., Klevit R.E. (1998). A disorder-to-order transition coupled to DNA binding in the essential zinc-finger DNA-binding domain of yeast Adr1. J. Mol. Biol..

[39] Dyson H.J., Wright P.E. (2016). Role of Intrinsic Protein Disorder in the Function and Interactions of the Transcriptional Coactivators CREB-binding Protein (CBP) and p300. J. Biol. Chem..

[40] Van der Lee R., Buljan M., Lang B., Weatheritt R.J., Daughdrill G.W., Dunker A.K., Fuxreiter M., Gough J., Gsponer J., Jones D.T. (2014). Classification of intrinsically disordered regions and proteins. Chem. Rev..

[41] Schaufler L.E., Klevit R.E. (2003). Mechanism of DNA binding by the Adr1 zinc finger transcription factor as determined by SPR. J. Mol. Biol..

[42] Haase S.B., Wittenberg C. (2014). Topology and control of the cell-cycle-regulated transcriptional circuitry. Genetics..

[43] Lieleg C., Krietenstein N., Walker M., Korber P. (2015). Nucleosome positioning in yeasts: methods, maps, and mechanisms. Chromosoma.

[44] Rudnizky S., Malik O., Bavly A., Pnueli L., Melamed P., Kaplan A. (2017). Nucleosome mobility and the regulation of gene expression: Insights from single-molecule studies. Protein Sci..

