# Supplementary material for: Protein Engineering of Multi-Modular Transcription Factor Alcohol Dehydrogenase Repressor 1 (Adr1p), a Tool for Dissecting In Vitro Transcription Activation
Source: Biomolecules. 2019 Sep 17;9(9):497. doi: 10.3390/biom9090497 (PMC6769490; doi:10.3390/biom9090497)
Supplement: Supplementary file 1 [file biomolecules-09-00497-s001.pdf]

Supplementary material for the manuscript:

Protein engineering of multi-modular transcription factor Adr1p (alcohol dehydrogenase repressor 1), a tool to dissect *in vitro* transcription activation

Memmo Buttinelli [#1], Gianna Panetta [#2], Ambra Bucci [1,2], Daniele Frascaria [1], Veronica Morea [3], Adriana Erica Miele [2,4]*

[1] Department of Biology and Biotechnology "Charles Darwin", "Sapienza" University of Rome, P.le Aldo Moro 5, 00185 Rome, Italy

[2] Department of Biochemical Sciences, "Sapienza" University of Rome, P.le Aldo Moro 5, 00185 Rome, Italy

[3] National Research Council of Italy (CNR), Institute of Molecular Biology and Pathology, P.le Aldo Moro 5, 00185 Rome, Italy

[4] present address: Insitut de Chimie et Biochimie Moléculaires et Supramoléculaires (ICBMS), UMR 5246 CNRS – UCBL - Université de Lyon, 43 boulevard du 11 Novembre 1918, 69622 Villeurbanne, France

[#]These authors equally contributed to the research work.

*Corresponding author: Department of Biochemical Sciences, "Sapienza" University of Rome, P.le Aldo Moro 5, 00185 Rome, Italy; adriana.miele@uniroma1.it; Tel.: +39-06-49910556

Supplementary Figure S1. Expression, purification, function and stability of miniADR1 constructs.

Supplementary Table S1. Sequences of oligonucleotides used as primers for PCR amplification of the mini-ADR1 constructs.

Supplementary Figure S1. Expression, purification, function and stability of miniADR1 constructs.

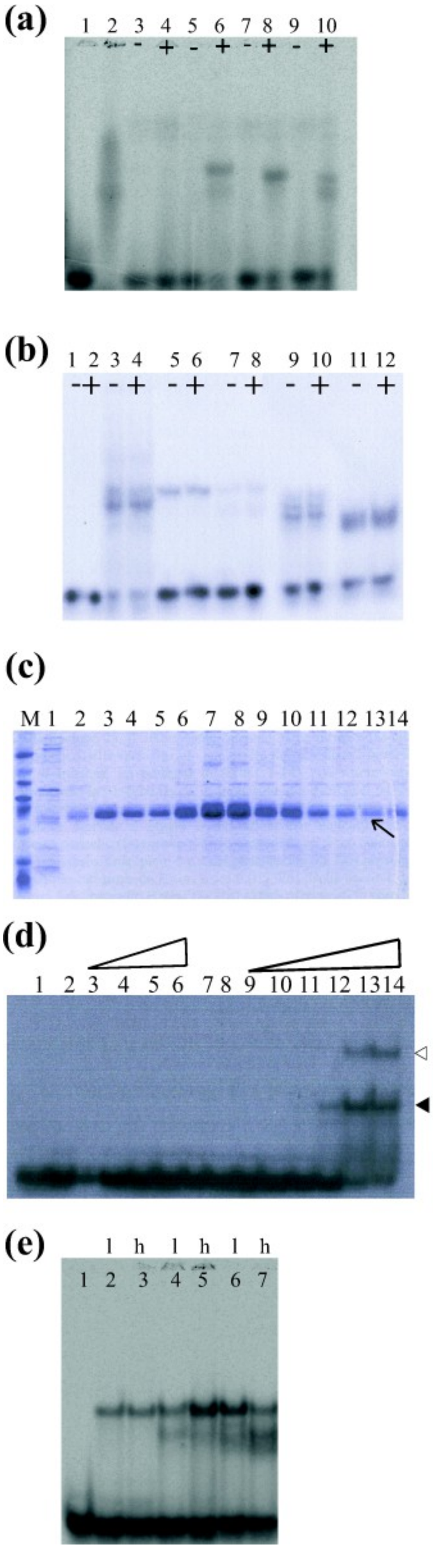

(a) Expression profiles of miniADR1-AL and miniADR1-BL in *Komagataella pastoris* (formerly classified as *Pichia pastoris*) with and without His-tag. Samples are cell lysates reacted with half-site radioactive DNA. Lane 1: free radioactive DNA probe. Lane 2: purified His-tagged miniADR1-BL from *E. coli* is shown for comparison purposes (see Material and Methods in the main text). Lanes 3-4: untransformed *K. pastoris* strain (negative control). Lanes 5-6: cells expressing His-tagged miniADR1-BL. Lanes 7-8: cells expressing untagged miniADR1-BL. Lanes 9-10: cells expressing untagged miniADR1-AL. (-) and (+) signs refer to cultures that were, respectively, not induced and induced for 48 h.

(b) Effect of dithiothreitol (DTT) on DNA-binding at different steps of untagged miniADR1-AL and miniADR1-BL purification. The probe is half-site radioactive DNA. Plus (+) and minus (-) signs indicate, respectively, the presence or absence of 5 mM DTT in the binding buffer at pH 7.5. Lanes 1-2 : *K. pastoris* cells expressing untagged miniADR1-BL lysed with glass beads. Lanes 3-4: *K. pastoris* cells expressing miniADR1-BL lysed with ultrasound. Lanes 5-6: purified untagged mini-ADR1-AL expressed in *K. pastoris*. Lanes 7-8: purified miniADR1-AL expressed in *K. pastoris* and stored at 4 °C for 3 weeks. These last were used as a control of stability/degradation of the purified protein. The reducing agent had no effect on binding; cell lysis by sonication degraded and reduced the amount of protein, as well as long term storage.

(c) 15% SDS-PAGE of His-ADR1-165 (*E. coli*) purification on Ni-binding column stained with Coomassie Blue. Lane M: MW marker. **Lane 1**: pool of proteins eluted from the heparin column and input of the Ni-NTA column. Lanes **2-14**: fractions eluted at 300 mM imidazole. His-ADR1-165 is indicated by the arrow; the purity does not exceed 80%.

(d) DNA binding assay of His-tagged ADR1-165 expressed in *E. coli* and revealed by N-PAGE using radioactive half site DNA probe. **Lanes 1-2** and **7-8**: free DNA probe. **Lanes 3-6**: titration of

the protein purified with CM sepharose, heparin and Ni-NTA affinity chromatography. **Lanes 9-14**: titration of the protein partially purified with CM sepharose and heparin. The dilution factor is 0.5 log per each lane. Lanes 6 and 14 contain 5 µl of undiluted sample. The white and black triangles indicate dimeric and monomeric DNA-protein complexes, respectively. The affinity chromatography step, despite a dialysis against Zn-rich buffer, abolished the DNA-binding. The cationic and heparin chromatography yielded a protein only about 40% pure, functional but unstable.

(e) DNA-binding assays of several purification steps of His-tagged miniADR1-BL expressed in *K. pastoris*. Lowercase "*l*" and "*h*" indicate low and high amount of loaded protein, respectively. **Lane 1**: free half site radioactive DNA probe. **Lanes 2-3**: cell lysates (4 and 8 µl). **Lanes 4-5**: protein fractions enriched in His-miniADR1-BL eluted from CM column were pulled together (1 and 2 µl). **Lanes 6-7**: protein fractions enriched in His-mini-ADR1-BL eluted from Q sepharose chromatography were pulled together (4 and 8 µl) (see also the manuscript). The protein was functional, but unstable, the purity did not exceed 80% and the final yield was also very poor (see Table 1 in the main text).

Table S1. Sequences of oligonucleotides used as primers for PCR amplification of the mini-ADR1 constructs. The oligo ADR1-75 contains an extra sequence for the restriction enzyme *Pml*I; oligos ADR1-157, ADR1-178, ADR1-401 and ADR1-419 contain the extra sequence for *BamH*I; the oligos ADR1-178 reverse-stop and ADR1-471 reverse contain *Apa*I restriction site, the stop codon and *Xho*I restriction site at the end of the gene sequence.

Oligonucleotide name	Sequence (5' → 3')
ADR1-75	TTGTCGCACGTGCATATGAACTCCAAGATTAACAAGCAACTG
ADR1-157 reverse	CGCGGATCCACTATGGATTTTTTGAG
ADR1-178 reverse	CGCGGATCCTTTCCGACGTTTAGTTATAGTTCTCG
ADR1-401	CGCGGATCCTGGACTGTTGCTATAGATAATAATAG
ADR1-419	CGCGGATCCCAACCTGATTTCGTCGATTTTC
ADR1-471 reverse	CCGCTCGAGCTATTAGGGCCCGTTCAAATGGGAAAGGTCAATC
ADR1-178 reverse with stop codon	CCGCTCGAGCTATTAGGGCCCGGATCCTTTCCGAGCTTTAG
